# “Mass casualty management (Rana Plaza Tragedy) in secondary military hospital-anesthesiologist experience: case study”

**DOI:** 10.1186/2054-314X-1-2

**Published:** 2015-01-27

**Authors:** Hasan Murshed, Rokshana Sultana

**Affiliations:** 1 Department of Anesthesiology and Intensive Care, Combined Military Hospital, Savar, Dhaka Bangladesh; 2 Mailing address; Combined Military Hospital Savar, Dhaka, Bangladesh

**Keywords:** Anaesthesiologist, Principal issues, Mass casualty

## Abstract

Major challenges in the management of mass casualty have been identified as lack of human resources, lack of material resources, lack of communication and co-ordination. Our hospital has limited resources of manpower and disposable items. The Departments of Anaesthesiology and Intensive Care have been seriously disrupted by the influx of 155 severely injured patients following the collapse of a nine storey building. Such a large, instantaneous influx of injured citizens would overwhelm even the most well resourced health care system.

A multidisciplinary team approach was planned to manage the casualties. Senior anaesthesiologists took responsibility for the organisation of different staff members into medical triage team, an immediate care team, an urgent care team, a non-urgent care team and a clerical team. Different teams have accomplished casualty management by addressing four principal issues (the assessment of available resources; ensuring critical but limited care; stocking up on medicine and equipment for the patient surge; and tough rationing of decisions).

Assessments of available resources were done by emphasising three #8216;S’s – staff (human resources), stuff (material resources) and structure. Additional human resources (anaesthesiologists, orthopaedic surgeons etc.) and material resources (#8216;H’ type oxygen cylinders, intravenous fluid etc.) were reinforced from nearby hospitals. Additional influxes of critical patients were supported in the postoperative ward and recovery rooms without any monitoring devices. A surgical dressing room without any basic monitoring device was used as an operating room. To do the greatest good for the greatest number of patients, we restricted ourselves to providing “essential rather than limitless critical care”.

“Stocking up on medicine and equipment resources” on assessment of the constraints in managing the patient surge, was the next essential step in the management of the casualty load. Patients with life-limiting illnesses were excluded from receiving scarce critical care resources. Thus “Tough rationing of decision” was also an important element.

Although the patients that were managed were not large in number, a consideration of the setup with a limited workforce and modern equipment and management experience of a mass casualty addressing the four principal issues in our department, might also help other departments in managing such events.

## Background

The worst factory disaster in the country's (Bangladesh) history occurred around 8:30 am on Wednesday 24 April 2013. A nine-storied commercial building “Rana Plaza” collapsed on the outskirts of the capital of Bangladesh. According to official reports 1,132 people were killed and 2,438 others injured in this disaster.

Mass casualty management is based on the specific training of various levels of responders and incorporation of links between different health care facilities through a command post. [1] It is recognised that a lack of human resources, lack of material resources, lack of communication and co-ordination provide significant challenges during mass casualty events [2–4]. Management of casualties is not infrequent in a hospital. But an established procedure to manage mass casualties in our department of Anaesthesiology and Intensive Care was lacking. This resulted in the disruption in our department with the influx of 120 severely injured patients within two hours of building's collapse. Such a large, instantaneous influx of injured citizens would overwhelm even the most well resourced health care system. We share the working experience of managing mass casualties using a multidisciplinary team and addressing four principal issues (assessment of available resources, ensuring critical but limited care, stocking up medicine and equipment for patient surge, tough rationing of decision) in our Department of Anaesthesiology and Intensive Care.

## Case presentation

In this study the clinical records of victims of the building collapse (Rana Plaza Tragedy) in Bangladesh were analysed retrospectively. This study was approved by our hospital authority. No informed consent was necessary as this study used existing data. Among 431 patients reported to the emergency and casualty department, only 155 (35.962%) were treated in the Department of Anaesthesia and Intensive care (Table 1). Most of the injuries were blunt trauma and soft tissue, the rest of the injuries were fractures, head injuries, crush injuries etc.

**Table 1:** **Patients influx at emergency department and admission in ICU (n =155)**

Day	Reported at emergency and casualty dept; n	Admitted in hospital: n (%)	Dead on arrival: n	Treated in ICU: n (%)
1.	122	112 (91.80)	10	34 (30.35)
2.	105	99 (94.28)	06	24 (24.24)
3.	130	126 (96.92)	4	39 (30.95)
4.	43	40 (93.02)	3	40 (100)
5.	17	16 (94.11)	1	08 (50)
6.	06	06 (100)	-	06 (100)
7.	04	04 (100)	-	00
8.	01	01 (100)	-	01 (100)
9.	02	02 (100)	-	02 (100)
10 to 16	Nil	Nil	Nil	Nil
17.	01	01 (100)	-	01 (100)
Total	431	407 (94.43)	24	155 (35.962)

Our hospital is with limited human and material resources. We had only two anaesthesiologists, two surgeons, one ICA (Intensive Care Assistants) and six OTA (Operation Theatre Assistants) to run ICU (Intensive Care Unit) and the OR (Operating Room) on a round the clock basis. We had only two functional operating rooms. Our ICU is four bedded with only one functioning ICU ventilator, without any other advanced organ support system (such as dialysis). Our hospital became extremely disorganised on the day of the building collapse with the influx of patients (Table 1). A multidisciplinary team approach was planned to provide the smooth care of this patient influx. Senior anaesthesiologists took the responsibility and organised different staff into different teams to share the responsibilities. Teams were organised into a medical triage team, an immediate care team, an urgent care team, a non-urgent care team, and a clerical team. One anaesthesiologist was constantly present in the emergency and casualty department along with one surgical specialist and medical specialist as medical triage team members for the quick assessment of patients. The most Senior anaesthesiologist and orthopaedic surgeons were involved in ICU and OR as immediate care team members for the treatment of life threatening injuries. One anaesthesiologist and one surgical specialist were responsible for first aid or evacuation of patients from the place of incident or hospital to another hospital as and when required as urgent team members. One anaesthesiologist and one surgical specialist were working in ICU and OR as non-urgent care team member for treatment of nonlife threatening injuries/illness. Clerical jobs such as documentation were assisted by military paramedics. The OTAs and ICAs were organised to run 3 surgical operative procedures simultaneously and continue care in ICU round the clock.

Preoperatively many patients were dehydrated or under resuscitated and were unable to speak. Thus medical history about previous medical disease, verification of fasting status, and inquiry about drug allergies were not possible in all patients. To avoid perioperative complications, all patients were considered as having hyper reactive airways and a full stomach before induction of anaesthesia. No drugs were administered without skin tests for sensitivity. Preoperatively 36 patients had Haemoglobin below 10.0gm per 100 ml and 24 patients had creatinine labels above 1.2 mg per deciliter. Anaemia was very common, possibly secondary to preexisting malnutrition and severe injuries with concomitant blood loss. Serum creatinine was also high in good number of patients, possibly due to associated acute kidney injury (AKI).

**Figure 1:**
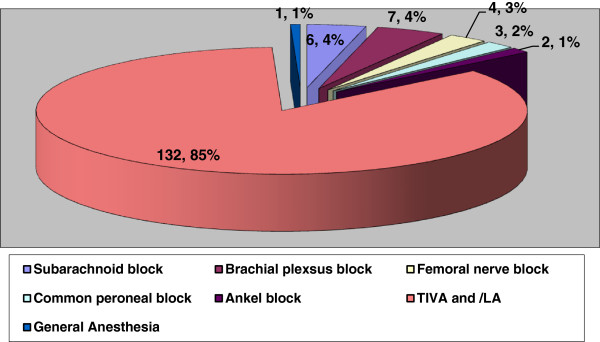
**Pattern of anesthetic procedures in different surgical procedure.**

Surgical procedures done under anaesthetic cover are presented in Table 2. The majority of surgical procedures were wound debridment, fascitomy, amputation and external fixation. Most of the operative procedures were done under total intravenous agent (TIVA) followed by different regional anaesthesia techniques (Figure 1). General anaesthesia was induced to only one patient who was requiring airway management. Among regional anaesthetic techniques, brachial plexsus block, peroneal nerve block, ankle block, spinal block etc. were performed without nerve stimulator and ultrasound. Postoperatively, three patients required the support of mechanical ventilation but due to the limitation of ventilators, all these patients were transferred to different hospitals for definitive treatment.

**Table 2:** **Surgical operative procedure done at combined military hospital**

Day	Operative procedure					
	Major procedure				Minor	Total
	Fasciotomy	Amputation	Wound debridment	External fixation of fracture	Dressing and/POP	
**One**	**1**	**-**	**6**	**-**	**9**	**16**
**Two**	**5**	**1**	**9**	**1**	**14**	**30**
**Three**	**3**	**1**	**6**	**-**	**10**	**20**
**Four**	**6**	**1**	**8**	**1**	**6**	**22**
**Five**	**-**	**-**	**2**	**-**	**-**	**2**
**Six**	**-**	**-**	**-**	**-**	**35**	**35**
**Seven**	**-**	**-**	**-**	**-**	**30**	**26**
**Total**	**15**	**3**	**31**	**2**	**104**	**155**

## Discussion and evaluation

Mass casualty in the Department of Anaesthesiology and Intensive Care was managed under the command of the Senior anaesthesiologist as an expert on critical care and was involved from on-the-spot first aid to intensive care unit and operating room trauma surgery. The Senior anaesthesiologist took responsibility to organise different staff members into different teams to share the responsibilities. Different teams accomplished their casualty management addressing the four principal issues in every step of casualty management. The first issue included “assessment of available resources”; second was “ensuring critical but limited care”; third was “stocking up medicine and equipment” for the patient surge and finally “tough rationing of decisions”.

Assessments of resources based on three #8216;S's staff, stuff (equipment, pharmaceuticals, and supplies), and structure (both physical structure and management infrastructure such as the Incident Command System) were the first step in mass casualty management. Lack of enough human and material resources to manage the casualty in our department was identified, thus additional resources from nearby hospitals were reinforced. Amy Kaji A et al. in their study demonstrated ensuring these three aforementioned essential components (staff, stuff, and structure) enhance patient-care capacity [5]. Human resources (e.g. anaesthesiologist surgeons, OTA and ICA etc.) and material resources (e.g.: #8216;H’ type cylinder for manifold of central oxygen supply, colloid, venous and urinary catheter etc.) were reinforced from nearby military hospital within 24 hours. We had only one functioning ICU ventilator, thus one available portable ventilator and anaesthetic breathing circuit were modified to serve a partial purpose of ventilator in ICU. To support the additional influx of critically ill patients, the post operative ward and recovery room were used to support them. To deal with the increased operative load, we modified the surgical dressing room into an operating room with no modern monitoring capability. Patients were assessed and monitored by traditional physical examination: chest excursion, colour of body, carotid pulse and manual blood pressure measurement. Thus timely arrangements of resources according to the anticipated number of casualties were one of the vital lessons learned in our casualty management.

A second issue was “essential rather than limitless critical care” to allow additional patients to have access to life sustaining intervention during mass casualty. The concept of minimal acceptable care is the key to a staged management approach during a mass-casualty incident as highlighted in the study of Hirshberg A et al. [6] So we have restricted ourselves in providing essential rather than limitless critical care, to “Do the greatest good for the greatest number of people”. Injuries that required prolonged definitive surgery were not performed in ourhospital, but referred to other hospitals. Moreover due to limitation in the number of ventilators in our hospital, all mechanically ventilated patients were also transferred to different hospitals. The lesson learned by ensuring “essential rather than limitless critical care” is that we were able to provide critical care support of 155 patients in our resources limited Department of Anaesthesia and Intensive Care, over a short time span.

"Stocking up medicine and equipment resources” to manage the patient surge was the third issue to consider. Current hospital reliance is on “just-in-time” and stockless material management systems to reduce storage and inventory costs [7]. It leaves institutions with vulnerably low reserves of disposable and durable medical equipment [8]. Critical care equipments are no exception. So with the influx of additional critically ill patients, a hospital cannot care without resupply. Thus proper assessment of constraint and ensuring resupply of both disposable and durable equipment is also essential in the management of patient surge after a mass casualty.

Finally “Tough rationing of decision” is the fourth issue in the management of our MCI. Thus patients with life-limiting illness (e. g: severe trauma, unwitnessed or witnessed events that don't respond to electrical therapy etc.) would be excluded from receiving scarce critical care resources. Simultaneously ethical commitment to alleviate discomfort without intentionally hastening death was also ensured. Here euthanasia is also not acceptable.

Most of the operative procedures were done under TIVA and regional Anaesthesia. It is well established that Ketamine is useful during minor procedures (such as wound debridement and painful dressing etc.), especially if there is no recovery ward and there is lack of trained anesthetists [9, 10]. This drug is found to be remarkably safe but not absolutely safe, so one has to be vigilant also. Eighty five percent of our patients received TIVA with ketamine for surgical procedure and recovery were uneventful. This strengthens the role of TIVA and regional anaesthesia in any mass casualty where there is scarcity of trained nursing staff in ORs and PACU (post anaesthesia care unit).

The philosophies of medical care in response to a mass casualty incident no longer revolve around the individual patient. Medical resources, personnel, supplies and facilities are carefully allotted to provide the greatest good to the greatest number. As we had no documented procedure for managing a mass casualty in our department, our work was based on literature, knowledge and past experience of casualty management. Lessons learned are the measures which allowed us to overcome difficulties during the management of casualty. Our mass casualty management approach using a multidisciplinary team, addressing four principal issues, may assist other departments.

## Conclusion

Anesthesiologists are well educated and experienced to manage surges in perioperative services. Anesthesiologists can improve patient care and hospital efficiency by optimizing facility utilization, surgical/anesthesia logistics, man power management, institutional communication, and leadership. Although patients managed by number are not large but considering setup with limited workforce and limited modern equipment; practical lessons learned from the present experience of working on four principal issue might help other departments in managing such events. A well placed mass casualty incident command system enhances the capacity for doing greater good for greater number of victims.

## Consent statement

This article on experience was approved by our hospital authority. No informed consent was necessary as this study used existing data. Patient’s identity was hidden. This study retrospectively investigated the clinical records of 155 patients treated in ICU and operation theater of combined military hospital Savar with building collapse-related injuries following 24th April 2013 “Rana Plaza Tragedy”.

## Abbreviations

ICA: Intensive care assistant
OTA: Operation theater assistant
ICU: Intensive Care Unit
OR: Operating room
CMH: Combined military hospital
PACU: Postanesthesia Care Unit
AKI: Acute kidney injury
MCI: Mass casualty incident
TIVA: Total intravenous anesthesia.
